# Supplementary material for: Ubiquitin and Ubiquitin-Like Proteins and Domains in Ribosome Production and Function: Chance or Necessity?
Source: Int J Mol Sci. 2021 Apr 22;22(9):4359. doi: 10.3390/ijms22094359 (PMC8122580; doi:10.3390/ijms22094359)
Supplement: Supplementary file 1 [file ijms-22-04359-s001.zip › SUPPL MARTIN VILLANUEVA ET AL/Suppl material Martin-Villanueva.pdf]

International Journal of
Molecular Sciences

MDPI

SUPPLEMENTARY MATERIAL

Ubiquitin and ubiquitin-like proteins and domains in ribosome production and function: chance or necessity?

Sara Martín-Villanueva [1,2], Gabriel Gutiérrez [2], Dieter Kressler [3,*] and Jesús de la Cruz [1,2,*]

1 Instituto de Biomedicina de Sevilla, Hospital Universitario Virgen del Rocío/CSIC/Universidad de Sevilla, Seville, Spain. saramarvi@msn.com (S.M.V.); ggpozo@us.es (G.G.); jdlcd@us.es (J.d.l.C.)

2 Departamento de Genética, Universidad de Sevilla, Seville, Spain.

3 Unit of Biochemistry, Department of Biology, University of Fribourg, Fribourg, Switzerland.

* Jesús de la Cruz, Instituto de Biomedicina de Sevilla, Campus Hospital Universitario "Virgen del Rocío". Avda. Manuel Siurot, s/n. E-41013, Sevilla, SPAIN. jdlcd@us.es; +34 955 923 126.

Dieter Kressler, Department of Biology, Unit of Biochemistry, University of Fribourg. Chemin du Musée 10. CH-1700 Fribourg, SWITZERLAND. dieter.kressler@unifr.ch; +41 26 300 86 45.

Table S1. List and taxonomic distribution of the 5349 proteins showing 80% identity over at least 80% of their length with the yeast ubiquitin domain. **See supplementary Table S1 excel file.**
Table S2. Architectures containing the ubiquitin domain. **See supplementary Table S2 excel file.**
Table S3. Rare variations of ubiquitin protein fusions.
Table S4. Proteins belonging to the Ubiquitin clan (CL0072) encoded by the *S. cerevisiae* genome as identified by PFAM. For more information and references about the function of the different proteins, readers are referred to the *Saccharomyces* Genome Database (SGD).

Figure S1. Number of pairwise yeast ubiquitin/PF00240 entry comparisons. The percentage shows the identity of the PF00240 architectural domain of all ubiquitin-containing proteins over at least 80% of their length with the yeast ubiquitin domain. Those proteins belonging to the PF00240 entry whose ubiquitin domain had less than 80% identity over at least 80% of its length with the yeast ubiquitin domain were discarded. In total, 5349 proteins were selected.

Figure S2. Schematic representation of the organization of different ubiquitin precursor proteins. Green blocks represent the ubiquitin sequence. The first amino acid and the di-glycine motif are shown. Generation of free ubiquitin from the different precursors requires the action of specific deubiquitinases (Dub) and the question mark denotes that the specific enzymes in charge of the cleavages are still unknown. (**A**) EhUbi1 is a single ubiquitin precursor corresponding to a monoubiquitin gene from *Entamoeba hystolytica*. Note that the ubiquitin moiety is synthesized as a precursor that contains a single extra amino acid, a tyrosine, that needs to be removed for ubiquitin activation. (**B**) Schematic representation of the four yeast ubiquitin precursors as a paradigm of the two classes of precursors found in most eukaryotes; Ubi1, Ubi2, and Ubi3 correspond to precursors containing a single copy of ubiquitin fused to the ribosomal protein eL40 (Ubi1 and Ubi2) and eS31 (Ubi3). Note that cleavage of the Ubi1 and Ubi2 precursors results in identical ubiquitin and eL40 proteins. Ubi4 corresponds a polyubiquitin precursor protein, which consists of a polymer of five tandem ubiquitin monomers. Note that the last monomer has an extra asparagine following the di-glycine sequence motif in the immature form.

Figure S3. Taxonomic distribution of unfused ribosomal proteins eS31 and eL40. The PFAM database was searched for isolated eS31 and eL40 ribosomal proteins that are not fused to ubiquitin or other domains. Note that most proteins being retrieved by this criterion belong to the Archaea domain, but also reasonable representation percentages were found in plants, animals, and fungi.

Table S3. Rare variations of ubiquitin protein fusions

Variation [1]	Abundance (%)
**Ub-eS31 variants**	**0.29**
Ribosomal_S8e ǀ Ubiquitin ǀ Ribosomal_S27	0.17
Ubiquitin ǀ Ribosomal_S27 ǀ Sec10	0.04
Ubiquitin ǀ Ubiquitin ǀ Ribosomal_S27	0.04
Fer2_2 ǀ FAD_binding_5 ǀ FAD_binding_5 ǀ CO_deh_flav_C ǀ Ald_Xan_dh_C ǀ Ald_Xan_dh_C2 ǀ Ald_Xan_dh_C2 ǀ Ubiquitin ǀ Ribosomal_S27	0.02
Ubiquitin ǀ Ribosomal_S27 ǀ Methyltransf_23	0.02
**Ub-eL40 variants**	**2.21**
zf-RRN7 ǀ Ubiquitin ǀ Ribosomal_L40e	0.84
KxDL ǀ Ubiquitin ǀ Ribosomal_L40e	0.41
Ubiquitin ǀ Ribosomal_L40e ǀ Ribosomal_L40e	0.26
eIF-5A ǀ Ubiquitin ǀ Ribosomal_L40e	0.13
Ubiquitin ǀ Ubiquitin ǀ Ribosomal_L40e	0.09
Ubiquitin ǀ Ribosomal_L40e ǀ Ubiquitin ǀ Ribosomal_L40e	0.06
ShK ǀ ShK ǀ Ubiquitin ǀ Ribosomal_L40e	0.04
V_ATPase_I ǀ Ubiquitin ǀ Ribosomal_L40e	0.04
ATP-synt_C ǀ ATP-synt_C ǀ Ubiquitin ǀ Ribosomal_L40e	0.02
Ubiquitin ǀ Ubiquitin ǀ Ubiquitin ǀ Ubiquitin ǀ Ubiquitin ǀ Ubiquitin ǀ Ubiquitin ǀ Ubiquitin ǀ Ubiquitin ǀ Ubiquitin ǀ Ubiquitin ǀ Ribosomal_L40e	0.02
Ubiquitin ǀ Ubiquitin ǀ Ubiquitin ǀ Ribosomal_L40e	0.02
Ubiquitin ǀ Ribosomal_L40e ǀ Yip1	0.02
Ubiquitin ǀ Ribosomal_L40e ǀ Abhydrolase_6	0.02
adh_short_C2 ǀ Ubiquitin ǀ Ribosomal_L40e	0.02
E1-E2_ATPase ǀ Ubiquitin ǀ Ribosomal_L40e	0.02
EF-hand_5 ǀ EF-hand_5 ǀ Ubiquitin ǀ Ribosomal_L40e ǀ EF-hand_7	0.02
Vps52 ǀ Ubiquitin ǀ Ribosomal_L40e	0.02
Ubiquitin ǀ Ribosomal_L40e ǀ JTB	0.02
EF-hand_5 ǀ EF-hand_5 ǀ Ubiquitin ǀ Ribosomal_L40e	0.02
GCR1_C ǀ Ubiquitin ǀ Ribosomal_L40e	0.02
Ubiquitin ǀ Ribosomal_L40e ǀ EF-hand_5 ǀ EF-hand_5	0.02
Ubiquitin ǀ Ribosomal_L40e ǀ TAXi_N ǀ TAXi_C	0.02
Cortexin ǀ Homeodomain ǀ TRAM_LAG1_CLN8 ǀ Ubiquitin ǀ Ribosomal_L40e	0.02
Cortexin ǀ Homeodomain ǀ Ubiquitin ǀ Ribosomal_L40e	0.02
TAXi_N ǀ TAXi_C ǀ Ubiquitin ǀ Ribosomal_L40e	0.02
**Ub-Ub tandem variations > 9 units**	**1.77**
Ubiquitin x 9	0.73
Ubiquitin x 10	0.37
Ubiquitin x 11	0.24
Ubiquitin x 12	0.22
Ubiquitin x 13	0.07
Ubiquitin x 14	0.06
Ubiquitin x 16	0.02
Ubiquitin x 18	0.02
Ubiquitin x 32	0.02
Ubiquitin x 46	0.02

[1] These are PFAM domains. Proteins are annotated as the PFAM entries. The entries Ribosomal_S8e, Ribosomal_S27, and Ribosomal_L40e correspond to ribosomal proteins eS8, eS31, and eL40, respectively. For further details, please see **Tables S1** and **S2.**

Table S4. Proteins belonging to the Ubiquitin clan (CL0072) encoded by the *S. cerevisiae* genome as identified by PFAM. For more information and references about the function of the different proteins, readers are referred to the *Saccharomyces* Genome Database (SGD).

Protein	PFAM Description	Function
**Processable Ub-like domain**		
Ubi1	Ubiquitin	Ub-ribosomal protein eL40A fusion protein
Ubi2	Ubiquitin	Ub-ribosomal protein eL40B fusion protein
Ubi3	Ubiquitin	Ub-ribosomal protein eS31 fusion protein
Ubi4	Ubiquitin	PolyUb precursor (5 head-to-tail repeats)
Rub1	Ubiquitin	Yeast NEDD8
Smt3	Ubiquitin	Yeast SUMO1
Hub1	Ubiquitin	Promotes splicing. Unclear covalent or non-covalent conjugation
Atg8	Atg8	Component of the autophagosome. Conjugated to phosphoethanolamine
Atg12	APG12	Component of the autophagosome. Conjugated to Atg5
Urm1	Urm1	Promotes thiolation of selected tRNAs
**Non-cleavable Ub-like domain**		
Rsa4	NLE	60S ribosomal subunit assembly factor. Rea1 binder
Ytm1	NLE	60S ribosomal subunit assembly factor. Rea1 binder
Ola1	YchF-GTPase_C	P-loop ATPase. Related to translation
Ylf2	YchF-GTPase_C	Unknown function. Similar to GTP-binding protein
Atg5	APG5	Involved in autophagy
Rbg1	TGS	GTP-binding protein. Related to translation
Rbg2	TGS	GTP-binding protein. Related to translation
Ths1	TGS	Threonyl-tRNA synthetase
Ubp15	USP7_ICP0_bdg	Deubiquitinase. Ub-specific protease of specific proteins
Ubp16	USP7-C2	Deubiquitinase. Anchored to the outer mitochondrial membrane
Rad23	Ubiquitin	DNA damage response. Subunit of NEF2
Dsk2	Ubiquitin	Spindle pole body duplication
Mdy2	Ubiquitin	Required for efficient mating
Alf1	Ubiquitin_2	Alpha-tubulin folding protein. Microtubule maintenance
Ubx2	Ubx	Interacts with Cdc48. Involved in ERAD[1]
Ubx3	Ubx	Interacts with Cdc48. Involved in clathrin-mediated endocytosis
Ubx4	Ubx/ TUG-UBL1	Interacts with Cdc48. Involved in ERAD
Ubx5	Ubx	Interacts with Cdc48.
Ubx6	Ubx	Interacts with Cdc48.
Ubx7	Ubx	Interacts with Cdc48. Paralog of Ubx6
Shp1	Ubx	Interacts with Cdc48. Involved in ERAD
Ste11	Ras_bgd_2	Signal transducing MEK kinase. Involved in pheromone response and pseudohyphal growth
Ste50	RA	Adaptor protein. Signal transduction
Mpe1	DWNN	Subunit of CPF cleavage and polyadenylation factor. E3 ligase
Esc2	Rad60-SLD	Diverse roles in DNA metabolism
Avo1	CRIM	Component of TORC2
Dsc3	DUF2407	Unknown function. Subunit of the DSC Ub ligase

[1]ERAD, ER-associated protein degradation

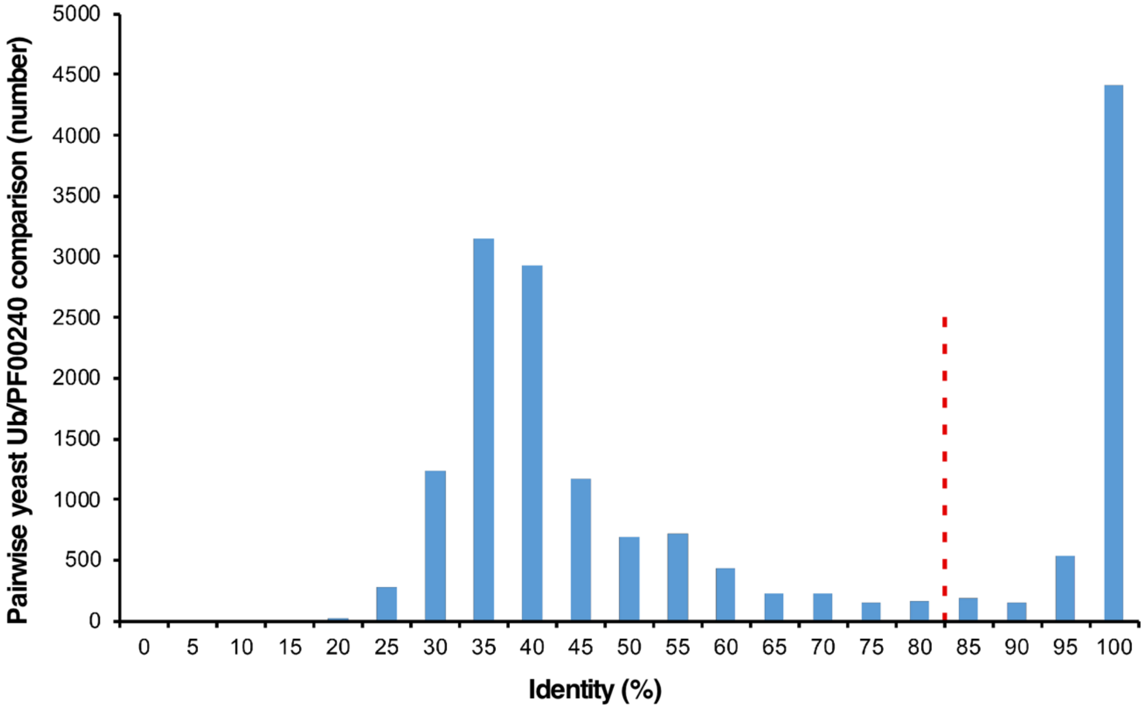

Figure S1. Number of pairwise yeast ubiquitin/PF00240 entry comparisons. The percentage shows the identity of the PF00240 architectural domain of all ubiquitin-containing proteins over at least 80% of their length with the yeast ubiquitin domain. Those proteins belonging to the PF00240 entry whose ubiquitin domain had less than 80% identity over at least 80% of its length with the yeast ubiquitin domain were discarded. In total, 5349 proteins were selected.

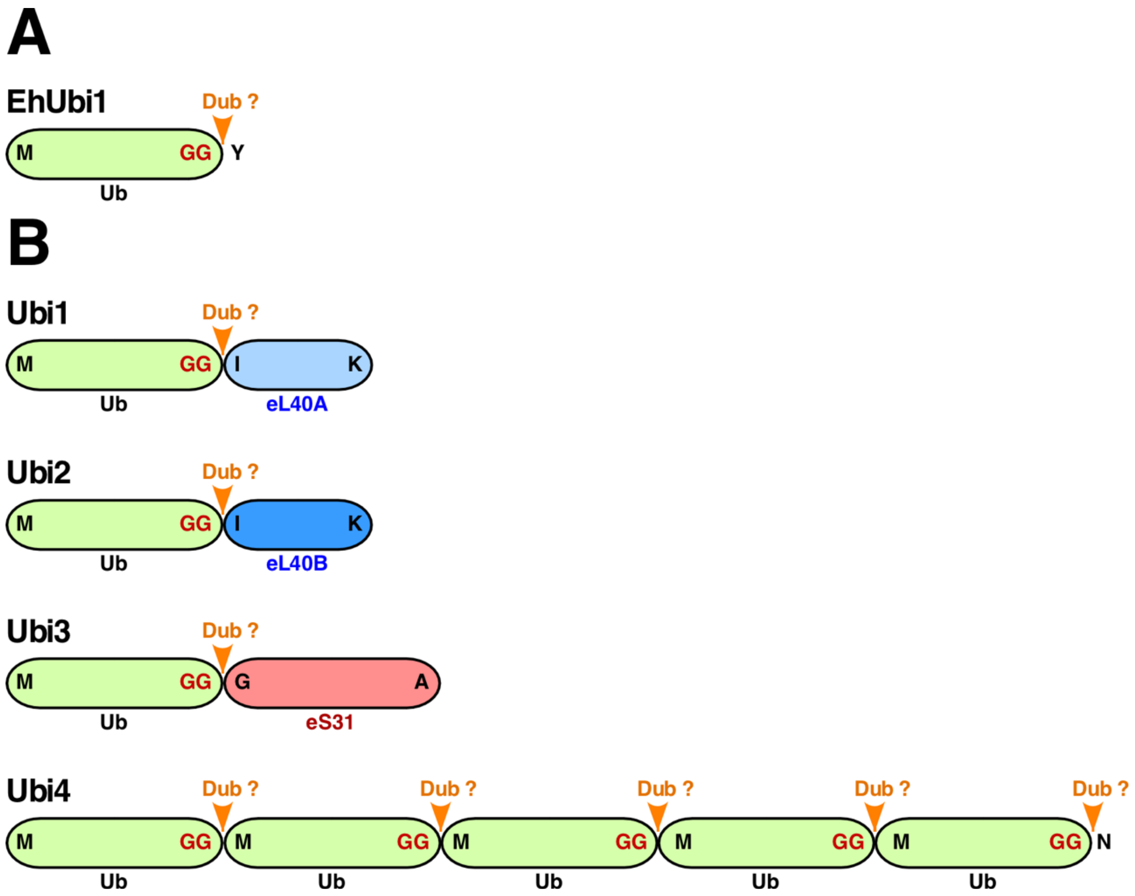

Figure S2. Schematic representation of the organization of different ubiquitin precursor proteins. Green blocks represent the ubiquitin sequence. The first amino acid and the di-glycine motif are shown. Generation of free ubiquitin from the different precursors requires the action of specific deubiquitinases (Dub) and the question mark denotes that the specific enzymes in charge of the cleavages are still unknown. (**A**) EhUbi1 is a single ubiquitin precursor corresponding to a monoubiquitin gene from *Entamoeba hystolytica*. Note that the ubiquitin moiety is synthesized as a precursor that contains a single extra amino acid, a tyrosine, that needs to be removed for ubiquitin activation. (**B**) Schematic representation of the four yeast ubiquitin precursors as a paradigm of the two classes of precursors found in most eukaryotes; Ubi1, Ubi2, and Ubi3 correspond to precursors containing a single copy of ubiquitin fused to the ribosomal protein eL40 (Ubi1 and Ubi2) and eS31 (Ubi3). Note that cleavage of the Ubi1 and Ubi2 precursors results in identical ubiquitin and eL40 proteins. Ubi4 corresponds a polyubiquitin precursor protein, which consists of a polymer of five tandem ubiquitin monomers. Note that the last monomer has an extra asparagine following the di-glycine sequence motif in the immature form.

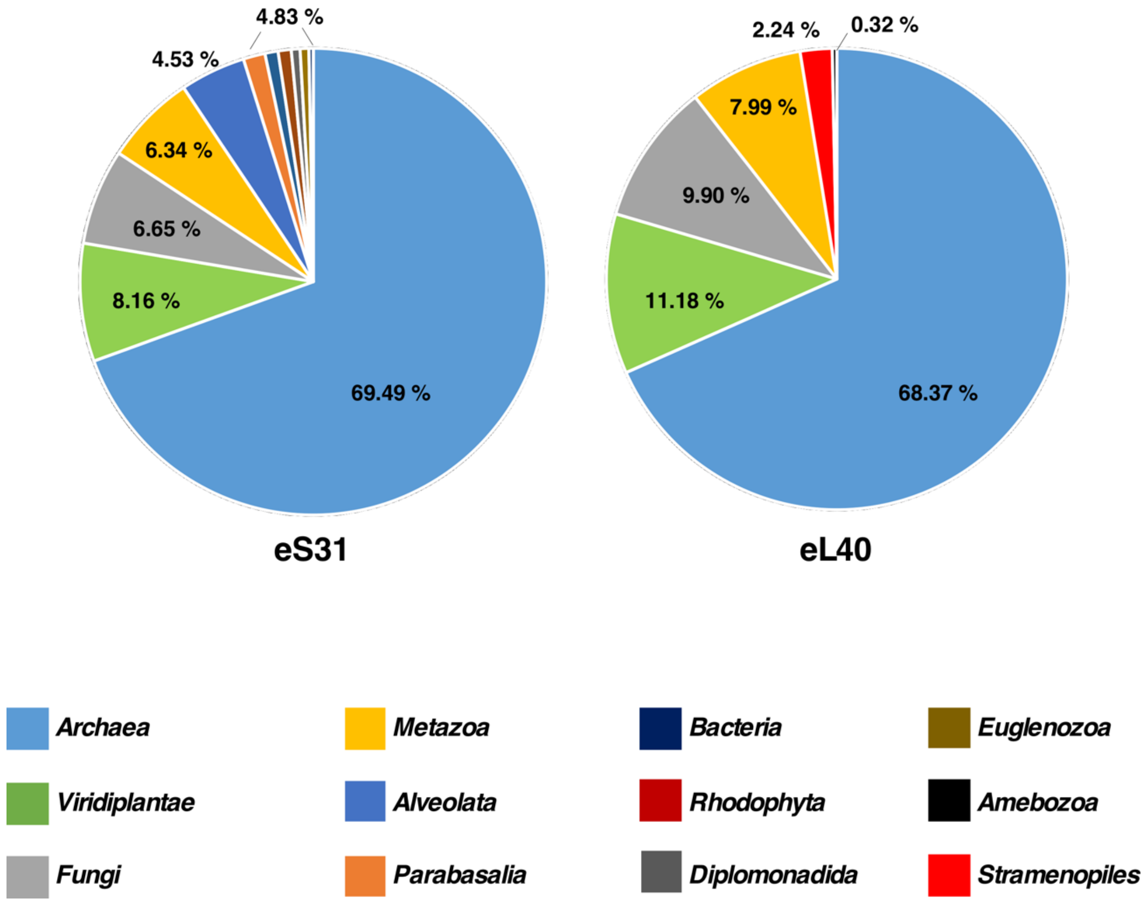

Figure S3. Taxonomic distribution of unfused ribosomal proteins eS31 and eL40. The PFAM database was searched for isolated eS31 and eL40 ribosomal proteins that are not fused to ubiquitin or other domains. Note that most proteins being retrieved by this criterion belong to the Archaea domain, but also reasonable representation percentages were found in plants, animals, and fungi.
